# Assessment of Ganglion Impar Block Effect on Treatment Results of Coccydynia: A Cross-Sectional Study

**DOI:** 10.5812/aapm-142137

**Published:** 2024-03-07

**Authors:** Aliakbar Nasiri, Farzad Farajzadeh Vajari, Shahryar Sane, Roghaiyeh Afsargharehbagh

**Affiliations:** 1 Department of Anesthesiology, Faculty of Medicine, University of Medical Sciences, Urmia, Iran; 2 Department of Cardilogy, School of Medicine, Urmia University of Medical Sciences, Urmia, Iran

**Keywords:** Coccydynia, Pain Management, Ganglion Impar Block, Treatment

## Abstract

**Background:**

The ganglion impar block is a minimally invasive technique used for alleviating pain associated with coccydynia.

**Objectives:**

This research evaluates the effectiveness of the ganglion impar block in treating patients with coccydynia who have not benefited from conservative treatments.

**Methods:**

This cross-sectional analysis reviewed the clinical records of coccydynia patients who received ganglion impar block injections at Urmia Imam Khomeini Hospital, Urmia, Iran, between 2020 and 2022. Data regarding age, gender, body mass index (BMI), onset of pain, and levels of patient satisfaction post-treatment were gathered from the medical records.

**Results:**

The study comprised 26 patients, with 4 (15.4%) being male and 22 (84.6%) female. The average age and BMI were 39.15 ± 14.24 years and 28.91 ± 2.14 kg/m^2^, respectively, which did not show significant variation (P = 0.19). The average Visual Analogue Scale (VAS) score before the ganglion impar block was 6.23 ± 2.35, which reduced to 4.47 ± 2.41 immediately after the procedure. At the 1-month follow-up, the average VAS score had further decreased to 3.47 ± 0.79. The decrease in VAS scores, both immediately after the procedure and at the 1-month follow-up, was statistically significant. The success rate of the block (defined as a reduction in pain of at least 20% from the baseline) was significantly high immediately and one-month post-procedure (P < 0.001). Out of the 26 patients treated with the impar block, satisfaction rates were 42.3% excellent, 27% good, 19.2% fair, and 11.5% poor.

**Conclusions:**

The study endorses the trans-sacro-coccygeal “needle inside needle” method for providing relief to patients suffering from coccydynia. The findings revealed significant patient satisfaction, with the majority describing their experience as excellent.

## 1. Background

Coccydynia, commonly referred to as pain in the tailbone, is a persistent pain condition that targets the coccyx, the small triangular bone at the bottom of the spine (1). This specific area is composed of approximately four undeveloped vertebrae that are connected to the sacrum, a connection first described by Simpson in the 19th century (2). The main cause of this pain is typically abnormal movement in the coccygeal area, which results in ongoing inflammation (3). Coccydynia tends to affect women more frequently than men and is often linked to being overweight (4). Although the condition most commonly appears around the age of 40, it can occur in people of all ages (5). The origins of coccydynia are diverse, including both physical and psychological factors. The leading physical causes are trauma from a fall onto the buttocks, repeated minor injuries, or childbirth, making these the most common triggers for coccydynia (6). Causes of coccydynia that are not related to trauma include degenerative diseases of joints or discs, unusual movement at the sacrococcygeal joint, obesity, infections, changes in the shape of the coccyx, or cancer in the pelvic region or anorectal (7).

The effectiveness of coccydynia treatments varies significantly (3). At present, it is not clear if the outcomes of these treatments are associated with specific patient characteristics (8). There are several conservative treatment options for coccydynia, such as non-steroidal anti-inflammatory drugs (NSAIDs), local analgesics, application of heat or cold, transcutaneous electrical nerve stimulation (TENS), use of specially designed wedge-shaped pillows (coccygeal cushions), and exercises to relax the levator ani muscle. These methods aim to alleviate pain and inflammation and enhance the functionality of the coccyx and its adjacent muscles (9). When these conservative approaches fail to provide relief, more invasive procedures may be considered. Techniques like injections of local anesthetics directly into the coccyx area, coccygeal nerve blocks, caudal epidural injections, and ganglion impar blocks offer minimally invasive alternatives for treating coccydynia (10). Coccygectomy, or the surgical removal of the coccyx, is an option for a limited number of patients (11). Surgical intervention, due to its potential for complications, is generally the last resort, considered only for those who have not benefited from both conservative and interventional treatments (12).

The ganglion impar (GI) is a unique retroperitoneal structure located at the sacrococcygeal junction, positioned variably within the pre-coccygeal space, marking the end of the bilateral sympathetic chains (13). It plays a role in providing nociceptive and sympathetic innervation to the perineum (14). When conservative treatments do not effectively relieve pain related to coccydynia, a GI block can be administered via the sacrococcygeal or intercoccygeal junction using imaging guidance such as fluoroscopy or ultrasound (15). The trans-sacrococcygeal technique for GI block, introduced by Wemm and Saberski in 1995 (16), aims to improve the ease of the procedure and reduce the risks of visceral damage that are possible with traditional methods. This technique is noted for being straightforward and quick to perform (17). Once the needle is accurately placed, local anesthetics, optionally combined with corticosteroids, are injected, and radiofrequency thermal ablation (RTA) can also be applied (14). However, there is scant clinical evidence to support the effectiveness of these procedures (18).

Few studies in existing literature (19, 20) have evaluated the effectiveness of GI blocks in managing pain. 

## 2. Objectives

This study sought to assess the effectiveness of GI blocks in treating coccydynia patients in Iran who have not benefited from conservative treatment approaches.

## 3. Methods

In this retrospective study, we examined the clinical records of coccydynia patients who underwent GI block treatment at Urmia Imam Khomeini Hospital in Urmia, Iran, from 2020 to 2022. We collected data on age, gender, body mass index (BMI), the onset of pain, and levels of patient satisfaction with the outcomes from the medical records. Pain severity was assessed using the Visual Analog Scale (VAS), a linear scale for pain evaluation that ranges from 0 (no pain) to 10 (the worst pain imaginable). This assessment was conducted before the procedure, immediately after, and one month following the injection. Patients were asked about their pain levels at these three time points, with the scores recorded on a predefined checklist. Patient satisfaction with the results was measured on a four-tier scale: Excellent, for complete relief or a reduction in pain of 75% or more; Good, for a pain reduction of 50% to 74%; Fair, for a reduction of 25% to 49%; and Poor, for a reduction of less than 25% or worsening of pain (3). The criterion for a successful block intervention was defined as achieving at least a 20% reduction in pain from the baseline. The ethics committee of Urmia University of Medical Sciences approved our study under the code IR.UMSU.REC.1397.483.

The study included participants who met specific eligibility criteria: They had been experiencing persistent coccyx pain for at least six months despite undergoing standard treatments and showed no abnormal lab or imaging findings that could explain their pain. Individuals were excluded if they had a local infection, a bleeding disorder, an allergy to contrast materials, or a history of spinal surgery. 

### 3.1. Ganglion Impar Block Procedure 

**Figure 1.**
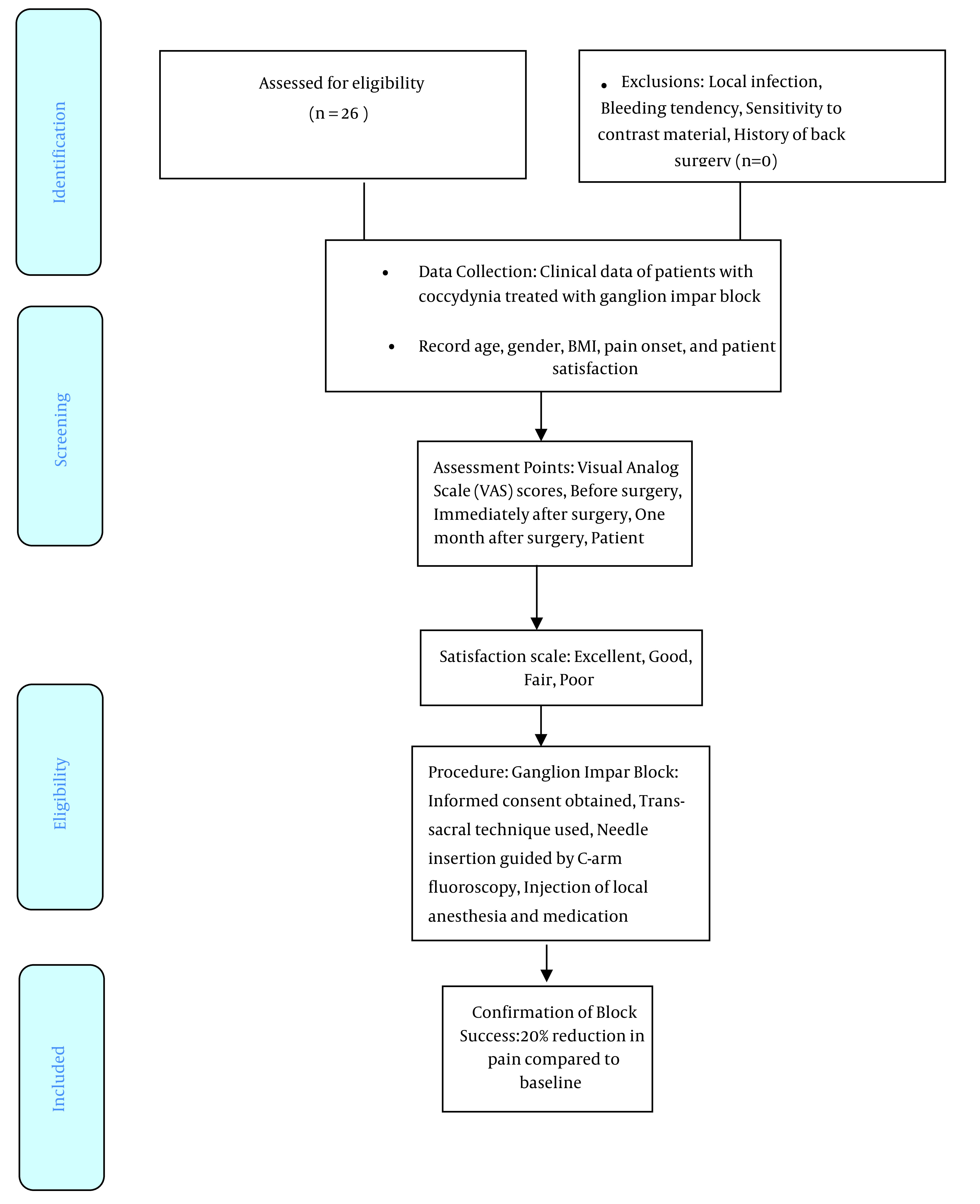
The patient selection process, intervention procedure, and assessment points.

The GI block procedure was conducted only after obtaining written informed consent from the patient, adhering to the ethical guidelines outlined in the Declaration of Helsinki. The trans-sacral approach was employed, involving the needle's insertion through the sacrococcygeal disc. For the procedure, the patient was positioned prone on the X-ray table, with a pillow placed under their lower abdomen for support. The injection site on the skin was cleaned, marked for needle entry, and then locally anesthetized. Under C-arm fluoroscopic guidance, a 22-gauge, 10-centimeter needle was precisely guided through the skin, piercing the dorsal sacrococcygeal ligament at the body's midline. The needle was then advanced through the intervertebral disc until it reached a position just in front of the ventral sacrococcygeal ligament, indicated by a reduction in resistance. To confirm the needle tip's accurate placement, 1 mL of radiopaque dye (Omnipaque 320 mg I/ml) was administered into the retroperitoneal space, with the lateral spread of the dye resembling a reversed comma shape, as illustrated in Figure 1. Following confirmation of the needle's position, a solution containing 5 mL of 0.5% bupivacaine and 40 mg of triamcinolone was injected. The patient's condition was closely monitored throughout the procedure for any signs of complications, with the entire process taking no more than 5 minutes to complete.

### 3.2. Statistical Analysis

Data were analyzed using SPSS Statistics version 20. Continuous variables are presented as means and standard deviations, while categorical variables are shown as frequencies and percentages. A repeated measures ANOVA test was utilized to assess the average pain levels before, immediately after, and one month following the block procedure. McNemar's test was applied to evaluate changes in recovery rates. A p-value of less than 0.05 was considered to indicate statistical significance.

## 4. Results 

This study comprised 26 patients who received the GI block treatment for pain management; among these, 4 (15.4%) were male and 22 (84.6%) were female. The overall mean age and BMI were 39.15 ± 14.24 years and 28.91 ± 2.14 kg/m^2^, respectively. The mean age for male patients was 30.50 ± 6.24 years, while it was 40.72 ± 14.80 years for female patients, showing no significant age difference between genders (P = 0.19). The mean BMI for male patients was 27.43 ± 2.11 kg/m^2^, and for female patients, it was 28.21±3.19 kg/m^2^, with no significant difference in BMI related to gender (P = 0.11) (Table 1). 

The mean VAS score prior to the GI block was 6.23 ± 2.35, which fell to 4.47 ± 2.41 immediately after the injection. At the one-month follow-up, the mean VAS score further decreased to 3.47 ± 0.79. The VAS scores were significantly reduced after the procedure and at the one-month follow-up. The success of the block (defined as a reduction in pain of at least 20% from the baseline) both immediately and one-month post-procedure in patients with coccydynia was statistically significant (P < 0.001) (Table 1). 

**Table 1.** Examination Findings and VAS Score Measurements of Patients with Coccydynia ^a^

Parameter	Values	P-Value
**Gender**		
Male	4 (15.4)	
Female	22 (84.6)	
**Mean age, year**	39.15 ± 14.24	0.19
Male	30.50 ± 6.24	
Female	40.72 ± 14.80	
**Mean body mass index, kg/m** ^**2**^	28.91 ± 2.14	0.11
Male	27.43 ± 2.11	
Female	28.21 ± 3.19	
**Mean Visual Analogue Scale score**		0.001
Before block	6.23 ± 2.35	
Immediately after block	4.47 ± 2.41	
One-month follow-up	3.47 ± 0.79	

^a^ Values are expressed as mean ± SD or No. (%).

According to Table 2, among the 26 patients who received the impar block, the levels of patient satisfaction were 42.3% excellent, 27% good, 19.2% fair, and 11.5% poor. 

**Table 2.** Satisfaction Ratio of Patients Undergoing Impar Block ^a^

Variable	Satisfaction				Total
	Poor	Faire	Good	Excellent	
**All Patients**	3 (11.5)	5 (19.2)	7 (27)	11 (42.3)	26

^a^ Values are expressed as No. (%).

## 5. Discussion

Coccydynia is characterized by various potential causes, and as of now, no definitive diagnostic criteria have been established (3). The GI, situated posterior to the peritoneum at the sacrum and coccyx junction, represents the terminal ganglion of the paravertebral sympathetic chain (21). In instances where conservative treatments do not yield results, a GI block is commonly considered as a viable treatment option for coccydynia (11). There are several methods for administering this block, which include using solely local anesthetics, combining local anesthetics with corticosteroids, applying neurolytic substances like alcohol or phenol to the nerves, and performing nerve destruction through RTA (22). We implemented a percutaneous minimally invasive technique using the GI block. Given its widespread availability and ease of use, we regard it as an effective and safe method for targeting the GI (23).

The treatment's effectiveness for chronic coccydynia patients correlated with changes in the VAS score (6). This condition is notably more common in females, with a ratio of five females to every male (24). While coccydynia can occur at any age, it is more prevalent in individuals aged 40 and older (14). Our patient cohort, which was 84.6% female with an average age of 39.15 ± 14.24, mirrors the findings of prior research (25). The increased incidence of coccydynia in women may be due to differences in pelvic anatomy. Research by Woon suggests that women's coccyxes are generally shorter and straighter, potentially making them more prone to retroversion (26). Furthermore, a higher BMI is recognized as a risk factor for coccydynia. In our study, the average BMI was 28.21 ± 3.19 kg/m^2^, nearing the obesity threshold (27).

Conservative medical treatments are effective in providing pain relief for the majority of coccydynia patients, as shown in a study (9). However, when the VAS score remains at 4 or higher despite conservative treatment, a GI block may be considered. In our research, the levels of patient satisfaction after undergoing the GI block varied, with 42.3% reporting it as excellent, 27% as good, 19.2% as fair, and 11.5% as poor. These findings are in line with Gonnade et al., who reported significant satisfaction with pain reduction among 31 patients following GI blocks over a 1-year follow-up period (20). Our study also noted a substantial decrease in pain scores: The average VAS score dropped from 6.23 ± 2.35 before the block to 4.47 ± 2.41 immediately after the injection and then to 3.47 ± 0.79 one month later. This trend of significant pain reduction parallels the findings of Sagir et al. and Gonnade et al., who observed a marked decrease in pain following a GI block in their 1-year follow-up studies (14, 20). Despite the broad success rate of 51 to 90%, it's important to acknowledge that GI blocks are linked with a high rate of complications and instances where pain relief was not achieved (28). Our data indicated a significant difference in the success rate of the ganglion block (defined as at least a 20% reduction in pain from baseline) immediately after the procedure and at the one-month follow-up. The precise identification of the ganglion's location is critical for the success of the block (29).

The widespread adoption of this highly effective block is constrained by notable complications, such as rectal perforation, hemorrhage, and infection, alongside the technical challenges it presents (18). To maximize safety and precision, we utilized fluoroscopy to verify the correct placement of the needle tip and the appropriate spread of the radiocontrast agent in the targeted area before conducting the blocks on all our patients. Fortunately, we did not encounter any complications during or following the procedures. Nevertheless, it is essential to recognize that while the GI block is generally safe, awareness of potential complications is crucial. The most frequent complications are temporary and minor, including pain increase at the injection site and vasovagal reactions. However, severe complications like rectum perforation, bleeding, infection, bladder incontinence, sexual dysfunction, and nerve root damage, although rare, can still occur (30, 31).

Acknowledging the study's limitations is vital for a balanced interpretation of the results. The small sample size of the study casts doubts on the applicability of the findings to a wider population. Moreover, the retrospective design of the study introduces possible biases, and the lack of a control group diminishes the capacity to make definitive statements about the procedure's effectiveness. Future research should aim for prospective, randomized studies with larger participant groups. Additionally, extending the follow-up duration from one month to six months could provide deeper insight into the intervention's long-term effects.

### 5.1. Conclusions

The results from this study indicate that the fluoroscopy-guided GI block is a promising approach for managing pain in coccydynia, showing signs of being safe, effective, and satisfactory to patients. This research proposes the GI block as a feasible method for alleviating pain in coccydynia, yet it emphasizes the need for further investigation and more extensive studies to confirm these preliminary findings and evaluate the long-term benefits and risks of the procedure.

## Data Availability

The dataset presented in the study is available on request from the corresponding author during submission or after publication.
